# Supplementary figures and images for: Ultrasound neuromodulation reveals distinct roles of the dorsal anterior cingulate cortex and anterior insula in learning
Source: PLoS Biol. 2026 May 5;24(5):e3003767. doi: 10.1371/journal.pbio.3003767 (PMC13143107; doi:10.1371/journal.pbio.3003767)

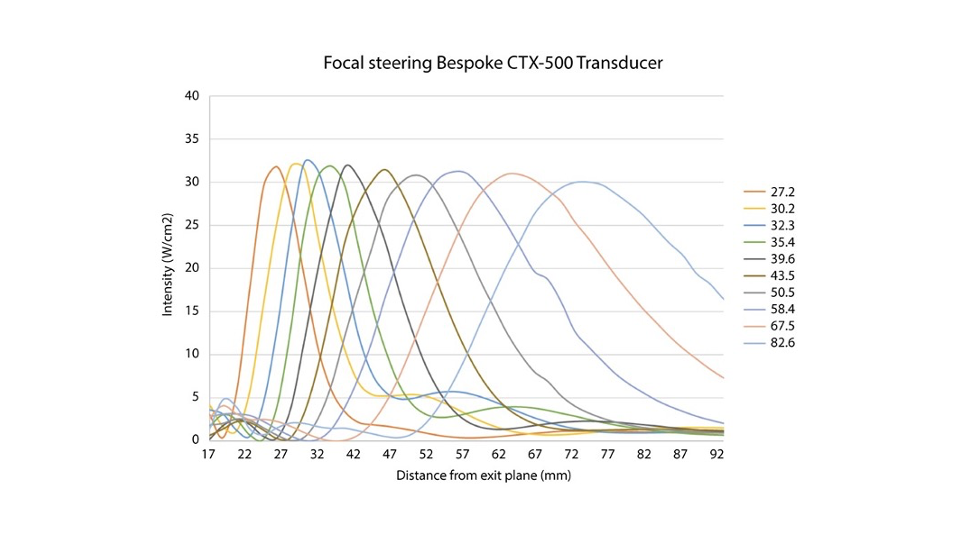

Supplement: S2 Fig — As the focus is steered axially, the intensity naturally decreases as the distance from the coherent focus increases. The TPO compensates for this decrease by adjusting the power, ensuring a consistent intensity level. Therefore, along the steering range from 27.3 to 82.6 mm (measured from the transducer’s exit plane), the power is regulated to maintain a constant ISPPA. The calibrated axial intensity profile plots are displayed in the figure at nine positions within the focal steering range from 27.3 to 82.6 mm. The figure is extracted from the manufacturer report. The range shown here is a calibration range used for this figure and does not represent the standard steering range of the CTX-500 system. (TIFF) [file pbio.3003767.s002.tiff]

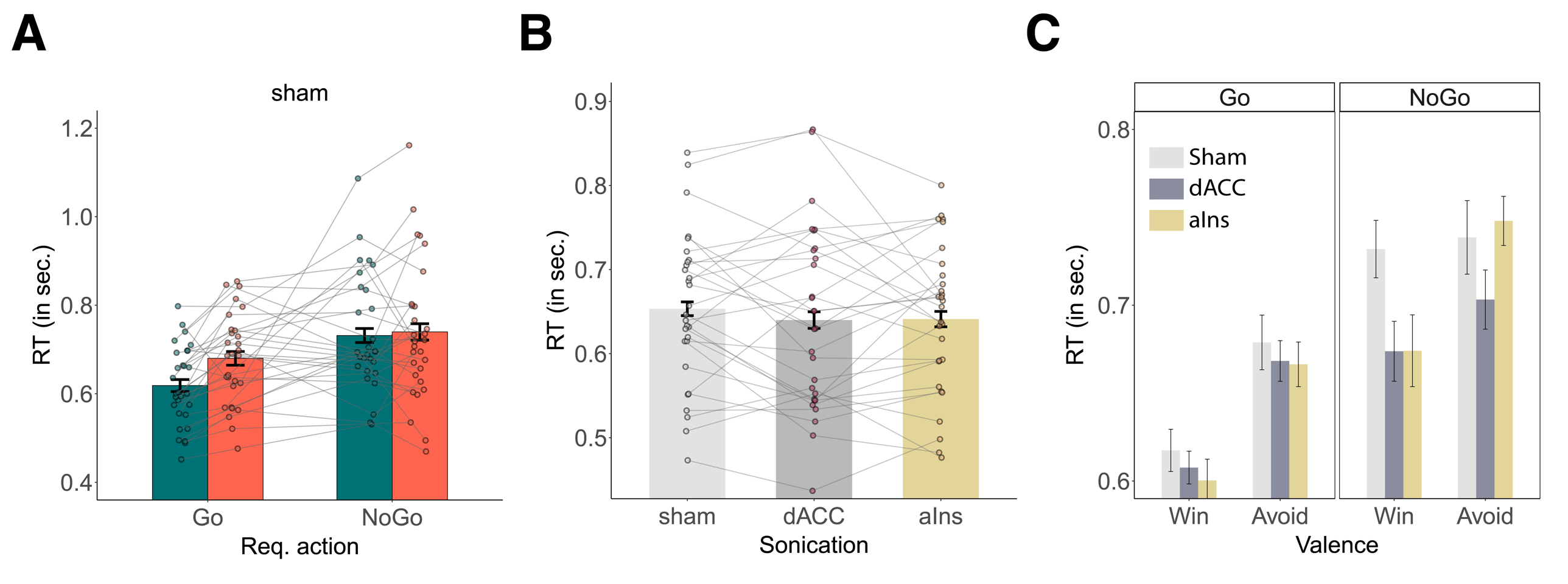

Supplement: S3 Fig — A. RTs for TUS-Sham split by required action (equivalent to accuracy given that RTs are only available for Go responses, and Go responses to Go cues are correct, Go responses to NoGo cues are incorrect) and cue valence. Participants showed faster RTs for (correct) responses to Go cues than for (incorrect) responses to NoGo cues, b = −0.207, 95% CI [−0.301, 0.113], χ2(1) = 18.505, p < .001 and for responses to Win than to Avoid cues, b = −0.081, 95% CI [−0.141, –0.021], χ2(1) = 6.927, p = .008, reflecting a Pavlovian response bias also in RTs. This bias was slightly stronger for Go than NoGo cues, b = −0.061, 95% CI [−0.107, –0.014], χ2(1) = 6.583, p = .010. B. RTs per sonication condition (Sham dACC, aIns). There were no differences in RTs between conditions, χ2(2) = 1.613, p = .446. C. RTs split by required action and valence for all three sonication conditions. The effect of required action on RTs was significantly stronger after TUS dACC compared to sham, b = −0.055, 95% CI [−0.099, −0.010], χ2(1) = 5.773, p = .016, with slower errors (responses to NoGo cues) after TUS dACC. Furthermore, the effect of cue valence on RTs was significantly stronger after TUS-aIns compared to sham, b = 0.037, 95% CI [0.002, 0.071], χ2(1) = 4.404, p = .036, driven by faster responses to Win cues after TUS-aIns. The data underlying this figure can be found in S5 Data. (TIF) [file pbio.3003767.s003.tif]

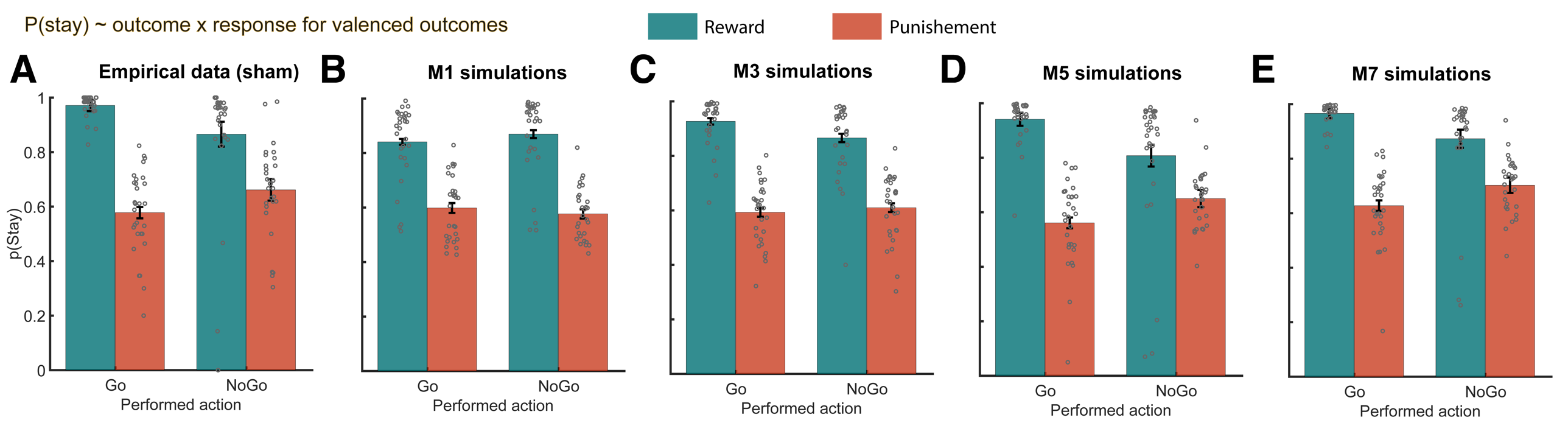

Supplement: S4 Fig — A. Probability of response repetitions given the previous outcome (reward vs. punishment; neutral outcomes omitted in this figure) and the previously performed action (Go vs. NoGo) for the sham condition of the empirical data. Participants show a stronger tendency to repeat responses after rewarded Go than rewarded NoGo responses, and a weaker tendency after punished Go than punished NoGo responses, reflecting a Pavlovian learning bias. B–E. One-step-ahead-predictions based on models M1, M3, M5, and M7. The asymmetry in outcome effects for Go and NoGo responses is only captured in M5 and M7 which feature a learning bias, but not in simpler models (M1, M3) without such a bias. The data underlying this figure can be found in S6 Data. (TIF) [file pbio.3003767.s004.tif]

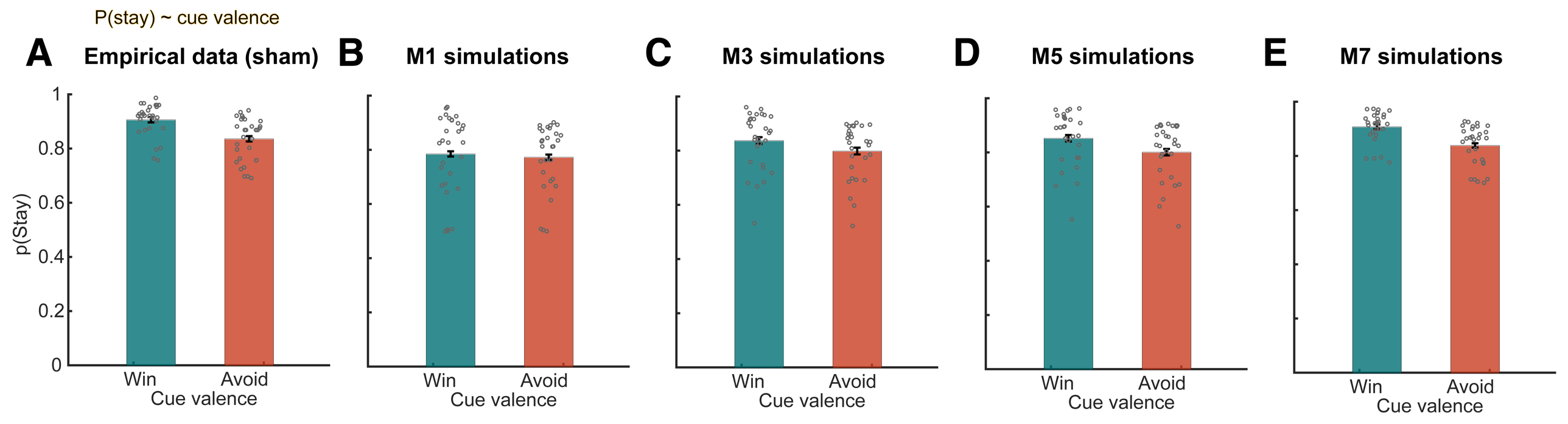

Supplement: S5 Fig — A. Probability of response repetitions given the cue valence (Win vs. Avoid) across all trials only in the sham condition. Participants show more response repetitions for Win than Avoid cues reflecting a Pavlovian persistance bias. B–E. One-step-ahead prediction based on models M1, M3, M5, and M7. The difference in response repetitions between Win and Avoid cues is only sufficiently captured by a model featuring a persistence bias (M7) seen in panel E, but less so or not at all by simpler models (M1, M3, M5), in panels B–D. The data underlying this figure can be found in S7 Data. (TIF) [file pbio.3003767.s005.tif]

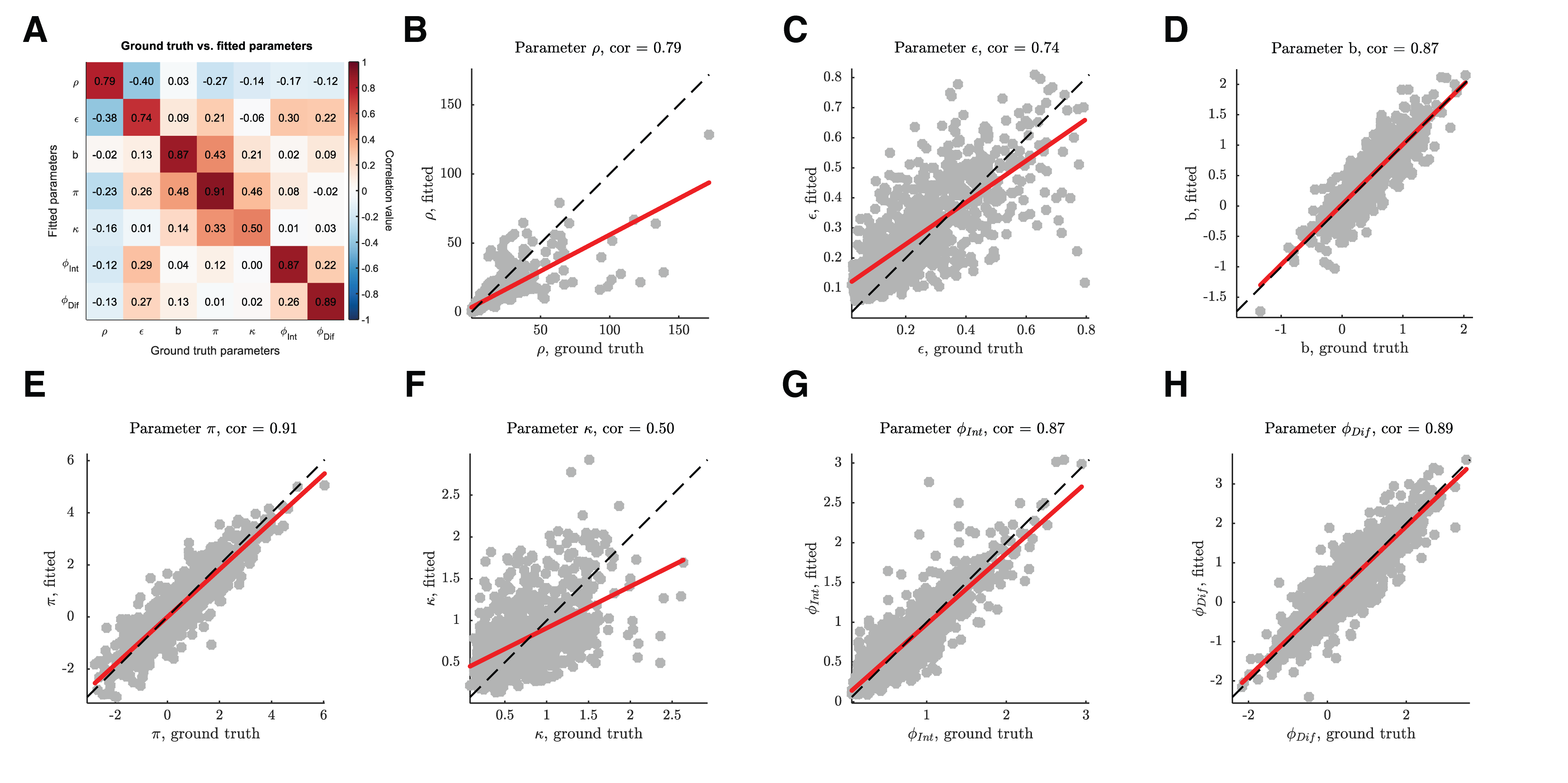

Supplement: S7 Fig — When simulating 1,000 new data sets from ground-truth parameters and fitting model M7 to these data sets, the fitted parameters correlate highly with the ground-truth parameters, demonstrating the ability of the model to reliably capture individual differences in parameters. A. Heatmap of correlations between ground truth (x-axis) and fitted (y-axis) model parameters. Parameters were well recoverable (on-diagonal correlations: range 0.50–0.91, median 0.86) with only small off-diagonal correlations (all < |0.43|). All on-diagonal correlations were significantly higher than expectable under a permutation null distribution (1,000 permutations; 95th percentile: 0.081). B–H. On-diagonal correlations for the feedback sensitivity (ρ), learning rate (ϵ), Go bias (b), Pavlovian response bias (π), Pavlovian learning bias (κ), persistence parameter (φINT), persistence bias (φDIFF) from simulated data. (TIF) [file pbio.3003767.s007.tif]

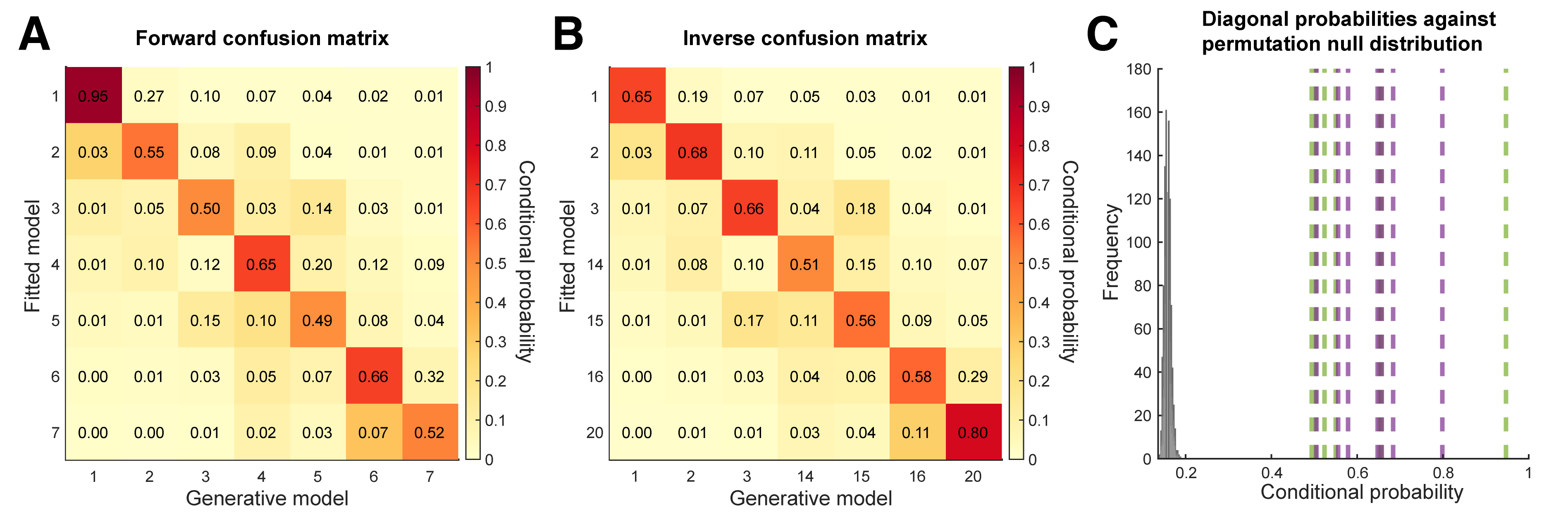

Supplement: S8 Fig — When simulating 1,000 new data sets from each model, fitting each data set with each model under consideration, and determining the best fitting model for each data set, the best fitting model most often corresponds to the original generative model, demonstrating the ability of reliably distinguish different models based on the data used in this experiment. A. Forward confusion matrix: A heatmap showing the conditional probability that data generated by a given model X (x-axis) is best fitted by model Y (y-axis). The diagonal elements show the probability of reidentifying the original generative model. All these probabilities are significantly higher than expected under a permutation null distribution (range 0.49–0.95, median 0.55; 95th percentile of permutation null distribution with 1,000 permutations: 0.171). B. Inverse confusion matrix: A heatmap displaying the probability that a data set best fitted by a given model X (x-axis) was in fact generated by model Y (y-axis). The diagonal elements show the probability that the best-fitting model is indeed the original generative model. All these probabilities are significantly higher than expected under a permutation null distribution (range 0.64–0.80, median 0.65; 95th percentile of permutation null distribution with 1,000 permutations: 0.171). C. Diagonal probabilities vs. permutation null distribution: The histogram displays the expectable on-diagonal conditional probabilities under a permutation null distribution (gray). Dashed vertical lines display the diagonal probabilities observed in the empirical confusion matrices (purple for inverse confusion matrix, green for forward confusion matrix), which are all higher than expectable under the null distribution. (TIF) [file pbio.3003767.s008.tif]

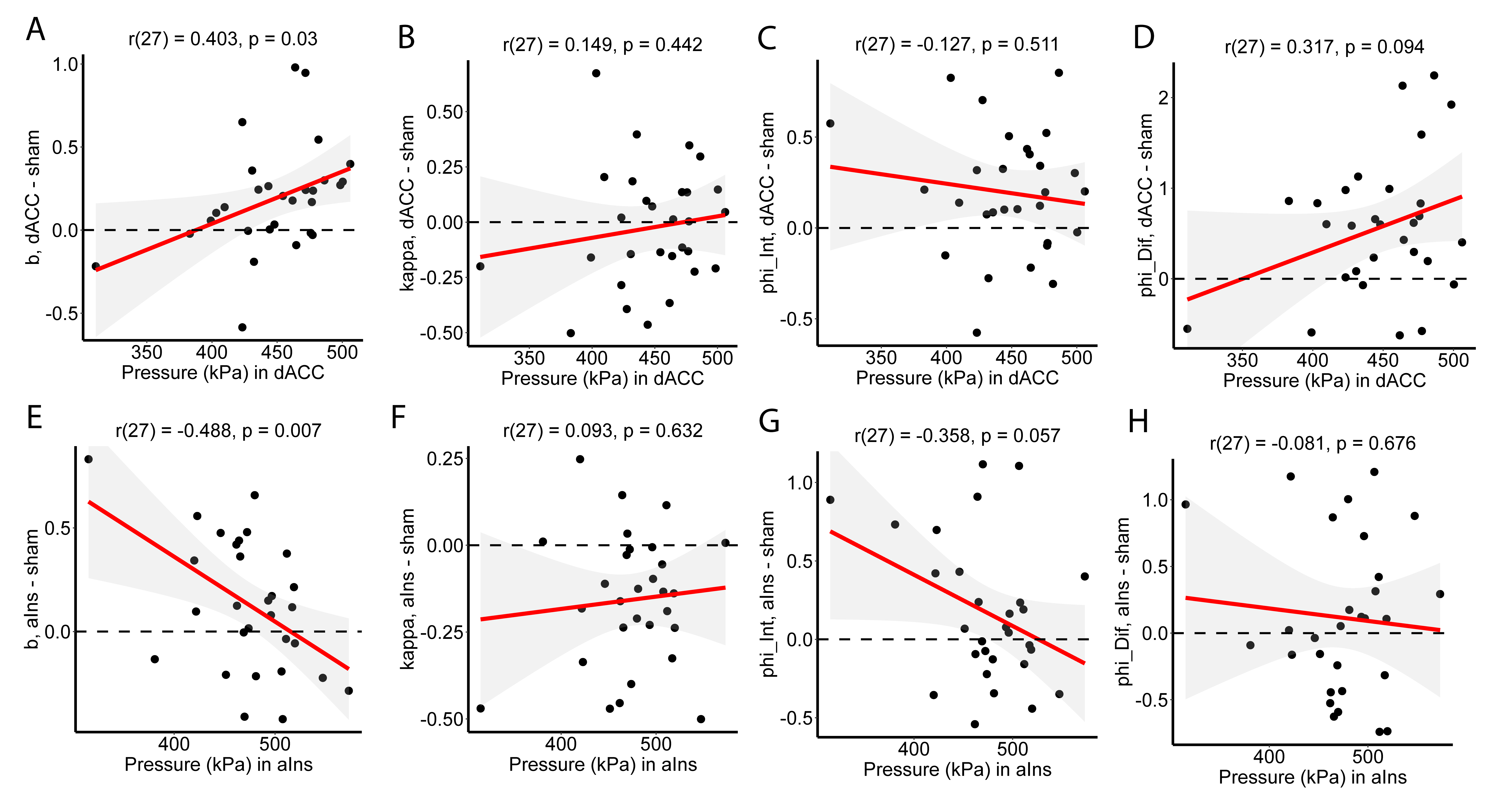

Supplement: S11 Fig — Scatterplots show the relationship between simulated in situ pressure (in kPa) for a given sonication condition (top row dACC, panels A–D; bottom row, aIns, mean of left and right aIns simulations, panels E–H) based on transducer and parameter coordinates recorded using Brainsight Neuronavigation during the sonications sessions, and changes in model parameters (b, κ, φINT, and φDIFF) between active stimulation and sham. Each point represents one participant. Red lines indicate least-squares regression fits and gray shading indicates 95% confidence intervals. Pearson correlation coefficients and two-tailed p-values (not corrected for multiple comparisons) are shown in the caption of each panel. Two associations reached nominal significance at the uncorrected threshold of p < 0.05 (A and E), but none survived correction for 8 tests (Bonferroni-corrected α = 0.00625). (TIF) [file pbio.3003767.s011.tif]
