# Supplementary material for: Ultrasound neuromodulation reveals distinct roles of the dorsal anterior cingulate cortex and anterior insula in learning
Source: PLoS Biol. 2026 May 5;24(5):e3003767. doi: 10.1371/journal.pbio.3003767 (PMC13143107; doi:10.1371/journal.pbio.3003767)
Supplement: S1 Appendix — (PDF) [file pbio.3003767.s015.pdf]

S1 Appendix
Adverse Events and Symptom Reporting Questionnaire

Absent = Not present

Mild = Present but not bothersome
Moderate = Tolerable - required some intervention/medication, but did not interfere with day-to-day activities
Severe = Intolerable - required contact with a GP or hospital A&E
Use the middle box to specify whether you think it might be related to the stimulation.
Use the box to the right of each symptom to provide details. e.g. describe what you felt, how long it lasted, medication you took to relieve the symptoms.

**Since your stimulation, have you had...?**	**Intensity of symptom**	**Relationship to stimulation**	**Please provide details**
a headache	absent	unrelated	
neck pain	mild	unlikely	
tooth pain	moderate	possible	
unusual feelings on your head or scalp	severe	probable	
itchiness		definite	
changes to your hearing			
speech problems			
vision problems (e.g. double vision)			
unusual twitching or muscle movement			
difficulties in balance			
changes in the movement of your strongest hand			
numbness or tingling sensations			
muscle tightness of the face or arm			
unusual feelings, attitudes or emotions			
anxiety, worried thoughts or nervousness			
increased sleepiness			
changes to your sleep pattern			
difficulty paying attention			
increased forgetfulness			
nausea or sickness to the stomach			
dizziness or light-headedness			
a seizure within the last 24 hours			
other symptom:			
other symptom:			
**Do you have anything else to report?**			
