# Supplementary material for: Ultrasound neuromodulation reveals distinct roles of the dorsal anterior cingulate cortex and anterior insula in learning
Source: PLoS Biol. 2026 May 5;24(5):e3003767. doi: 10.1371/journal.pbio.3003767 (PMC13143107; doi:10.1371/journal.pbio.3003767)
Supplement: S2 Appendix — (PDF) [file pbio.3003767.s016.pdf]

S2 Appendix
Contraindications and Safety Exclusion Criteria

Contraindications

1. Contraindications to Magnetic Resonance Imaging (MRI)

Metal Implants (pacemakers, cochlear implants, aneurysm clips)
Metallic fragments in the body
Tattoos or non-removable paramagnetic piercings on the neck or head.
Severe Claustrophobia
Pregnancy

2. Contraindications to Transcranial Ultrasound Stimulation (TUS)

2.1 Risk Groups and Vulnerable Populations - Pregnancy

Although magnetic fields (e.g., in repetitive transcranial magnetic stimulation (rTMS) attenuate quickly with distance and have been safely used in pregnant women (Klírová et al., 2008), no TUS studies have been conducted in this population.
Safety Strategy: Pregnant individuals will be excluded from all TUS studies until peer-reviewed research confirms safety in this group. If inclusion is necessary, it must be justified with specific ethical approval. Children and Adolescents

Despite the safe application of TMS in > 1100 paediatric cases (Frye et al., 2008), the developing brain is physiologically and pharmacologically different from adults.
Safety Strategy: Participants under the age of 18 will be excluded from TUS studies. Inclusion of minors would require specific ethics committee approval and scientific justification.

2.2 Pre-existing Medical Conditions

TUS-induced neural perturbation may be less predictable in participants with existing neurological disorders, posing additional risk.
Exclusion Criteria (Medical History)
Participants with the following conditions will be excluded due to heightened seizure risk or unpredictable neuronal(Rosa et al., 2004):

- Epilepsy or a first-degree relative with idiopathic (non-acquired) epilepsy
- Brain tumour
- Stroke
- Meningitis
- Encephalitis
- Severe head trauma Safety Strategy:
- All participants will be screened for these conditions using a detailed medical questionnaire.

Individuals meeting any of these criteria will not be enrolled.

2.3 Medications and Other Drug Use

The effect of TUS on individuals taking medication remains uncertain, particularly due to the potential interaction with the blood-brain barrier (BBB) or lowered seizure thresholds.
Note: TUS protocols used in our studies do not open the BBB. However, we adopt a cautious approach.

General Rule

Participants currently taking any medications will be excluded, including prescription, over-the-counter, or recreational drugs.
Participants must abstain from alcohol for at least 24 hours before the stimulation session.

High-Risk Drug Categories (based on rTMS seizure risk)
Category 1 – High Seizure Risk (strong exclusion):

- Tricyclic antidepressants (e.g., amitriptyline, imipramine)
- Antipsychotics (e.g., clozapine, chlorpromazine)
- Stimulants and illicit substances (e.g., amphetamines, 3,4-methylenedioxymethamphetamine
- (MDMA), cocaine, ketamine, phencyclidine (PCP), gamma-hydroxybutyrate GHB)
- Certain antivirals and antibiotics (e.g., ganciclovir, ritonavir, foscarnet, theophylline) Category 2 – Moderate Seizure Risk (relative exclusion):
- SSRIs (e.g., fluoxetine, sertraline, paroxetine)
- Anticonvulsants and immunosuppressants (e.g., cyclosporine, lithium)
- Antihistamines, sympathomimetics, anticholinergics
- Antimalarials (e.g., chloroquine, mefloquine) Category 3 – Withdrawal

Risk:

- Barbiturates
- Benzodiazepines
- Meprobamate
- Chloral hydrate

Safety Strategy:

- Participants taking or recently withdrawing from any of these substances will be excluded.
- Medication status will be assessed via screening forms prior to participation.

2.4 Seizure Risk

General Considerations
Although TUS has not been associated with seizure induction, safety measures are informed by the more extensive rTMS literature, where seizures have occurred, especially under high-frequency protocols or in combination with other risk factors (Conca et al., 2000; Rosa et al., 2004; Rossi et al., 2009).
Risk factors from these reports include:

- Use of pro-convulsant drugs (e.g., fluoxetine)
- Sleep deprivation
- History of epilepsy or brain injury

Safety Strategy for Seizure Prevention:

Individuals with seizure disorders or those at increased risk due to medication or medical history will be excluded.
All TUS protocols will strictly adhere to conservative safety parameters, and participants will be monitored throughout stimulation.

References

Conca, A., König, P., & Hausmann, A. (2000). Transcranial magnetic stimulation induces ‘pseudoabsence seizure’. *Acta Psychiatrica Scandinavica*, *101*(3), 246–248; discussion 248-249.

Frye, R. E., Rotenberg, A., Ousley, M., & Pascual-Leone, A. (2008). Transcranial Magnetic Stimulation in Child Neurology: Current and Future Directions. *Journal of Child Neurology*, *23*(1), 79–96. https://doi.org/10.1177/0883073807307972

Klírová, M., Novak, T., Kopecek, M., Mohr, P., & Strunzova, V. (2008). Repetitive transcranial magnetic stimulation (rTMS) in major depressive episode during pregnancy. *Neuro Endocrinology Letters*, *29*, 69–70.

Rosa, M. A., Odebrecht, M., Rigonatti, S. P., & Marcolin, M. A. (2004). [Transcranial magnetic stimulation: Review of accidental seizures]. *Revista brasileira de psiquiatria (Sao Paulo, Brazil : 1999)*, *26*(2), 131–134. https://doi.org/10.1590/s1516-44462004000200011

Rossi, S., Hallett, M., Rossini, P. M., & Pascual-Leone, A. (2009). Safety, ethical considerations, and application guidelines for the use of transcranial magnetic stimulation in clinical practice and research. *Clinical Neurophysiology : Official Journal of the International Federation of Clinical Neurophysiology*, *120*(12), 2008–2039. https://doi.org/10.1016/j.clinph.2009.08.016
