# Supplementary material for: Ultrasound neuromodulation reveals distinct roles of the dorsal anterior cingulate cortex and anterior insula in learning
Source: PLoS Biol. 2026 May 5;24(5):e3003767. doi: 10.1371/journal.pbio.3003767 (PMC13143107; doi:10.1371/journal.pbio.3003767)
Supplement: Attachment — Submitted filename: Response to reviewers_PLOSbiology_LAST_JA.docx [file pbio.3003767.s026.docx]

REVIEWER COMMENTS

**Reviewer #1 (Remarks to the Author):**

This study uses transcranial ultrasound stimulation (TUS) in a within-subject, counter-balanced, single-blind design (N=29; sessions: sham, dACC, aIns) to probe causal contributions of dACC and anterior insula to Pavlovian learning biases during a motivational Go/NoGo task. Behavior and RL modelling indicate a double dissociation: TUS-aIns attenuates a learning bias (credit assignment asymmetries), whereas TUS-dACC increases a persistence bias (cue-valence-dependent repetition), with some non-specific increases in Go bias/persistence under both active conditions. Overall, this is an innovative and rigorous study. A few clarifications are needed especially around targeting, dose modelling, sham blinding, and non-specific effects prior to publication.

We thank the reviewer for the thoughtful and encouraging evaluation of our study. We appreciate the recognition of the rigor and the novelty of the work, and we are grateful for the constructive suggestions that helped us improve clarity around targeting, modelling choices, and interpretation of nonspecific effects. Below we address each point in detail.

Major Comments

1) The central claim is a clean double dissociation yet both active conditions elevate overall Go bias and persistence relative to sham, suggesting a shared non-specific component (e.g., arousal/alerting, expectancy, auditory/somatosensory factors). To better unpack this please quantify effect sizes and confidence intervals for b and φ_INT across conditions, and state whether multiplicity-corrected results change interpretation. Suggestions could be to re-fit a model including a session-wise "arousal/alerting" nuisance component, or use mixed models to partial out non-specific changes before testing κ and φ_DIFF contrasts.

This is an important point that we have spent time considering. For overall Go bias, both active TUS conditions showed a similar, non-significant trend relative to sham after correction for multiple comparisons. At the same time, the region-specific effects of TUS-aIns on learning bias and TUS-dACC on perseveration bias remained significant, preserving a double dissociation. Although the absence of corrected effects on overall Go bias does not contradict, and could in principle strengthen, the double dissociation claim, we have nevertheless adopted a more conservative framing in the revised manuscript.

Accordingly, in the abstract, introduction, and throughout the results and discussion, we describe the pattern as a “functional differentiation” and “relative predominance of condition-specific effects”, rather than a strict double dissociation, in the context of broader but non-significant shifts that are shared across active stimulation sessions.

As requested, we now report effect sizes and bootstrapped confidence intervals for all model parameters. We agree that applying a correction for multiple comparisons is appropriate and therefore used a Bonferroni adjustment by dividing α = .05 by 7 (seven parameters, thus seven tests, new critical alpha-level: α = .007). Even under this conservative correction, the region-specific effects on κ and φ_DIFF remain significant (while unspecific effects on b and φ_INT are not significant, as already stated in the previous version of the manuscript), and our interpretation is unchanged. A dedicated paragraph describing these procedures and results has been added to the revised manuscript in page 14, line 530.

Regarding the reviewer’s suggestion to refit a session-wise "arousal/alerting" nuisance component, we are not sure what exactly the reviewer has in mind. Specifically, we are not sure how the reviewer expects high vs. low arousal to affect behaviour in this task. If arousal would impact the overall probability of Go responses or the overall probability of repeat responses, then b (Go responding bias) and φ_INT (repeat bias) are exactly such “nuisance components” and already present in the model.

Furthermore, we are not sure what the author means with a “session-wise [..] component”. A separate parameter for each session (i.e., 29 participants x 3 sessions = 87 more parameters) would obviously overfit the model and prevent us from assessing sonication effects. A separate parameter for each session number (i.e., 1st, 2nd, 3rd session) will reflect practice effects (see below). When controlling for such practice effects, sonication effects persist (see below).

Finally, regarding the reviewer’s suggestion to use mixed models to partial out nonspecific effects, we now performed such models testing for an effect of sonication on κ and φ_diff while controlling for go bias and perseverance intercepts’ parameter values in each respective session. The results are unchanged. We have now added this in the results section on page 14 in lines 538-:

“Notably, stimulation effects on κ and φINT were still significant when controlling for b and φINT, suggesting that region-specific effects occurred independently of these unspecific effects.”

Overall, we conclude that region-specific effects (on κ and φ_DIFF) are independent of unspecific effects (on b and φ_Int), which we now added in the Discussion section in page 17. We are grateful for the suggestion, which helped strengthen the manuscript.

2) You could report Bayes factors (or equivalently robust CIs) for the absence of κ change under dACC and of φ_DIFF change under aIns to strengthen the dissociation claim. However, the regression breakdowns (in Supplementary Data) reveal that both active conditions (dACC and aIns) produced effects across multiple cue types that are often marginally significant and overlapping. This weakens the claim of a clean double dissociation and suggests possible shared, non-specific effects. Unless anatomical targeting can be demonstrated at subregional precision (see below), functional interpretations should be tempered to emphasize relative predominance rather than strict dissociation.

As this point is addressed more fully in our response to the first comment, we have now tempered the strength of the dissociation claim throughout the revised manuscript. We therefore present the results in terms of relative predominance rather than a strict double dissociation. Also, we report robust bootstrapped CIs for all model parameters throughout. Please note that the changes in κ and φ_DIFF will, by their very nature, show up across multiple cue types. Analyses of Go/NoGo responses are appropriate for isolating the response bias π, but not κ and φ_DIFF, for which analyses of repeat/switch choices are more appropriate. We thus report both. The absence of strong effects on the analyses of Go/NoGo responses corroborates the absence of effects on π.

3) Because *in vivo* pressure differed between subjects between conditions this needs to be accounted for. You have individualized simulations and group summaries of dose. Please exploit this by relating per-subject in situ dose (e.g., on-target P_neg, I_SPPA) to change parameters (Δκ, Δφ_DIFF, Δb, Δφ_INT vs sham). Even null correlations will bound dose-response interpretations and bolster causal specificity.

We agree that variability in *in vivo* pressure across subjects and conditions is an important factor to consider. Using the individualized simulations, we examined the relationship between each participant’s estimated in situ dose (pressure in kPa, intensity in W/cm2) and the corresponding changes in modelling parameters relative to sham (Δκ, Δφ_diff, Δb, Δφ_int). These analyses did not show any significant relationship between pressure (or intensity) and the model parameters and therefore we decided not to report it in the paper.

It is important to note that amplitude estimates from numerical simulations carry substantial uncertainty. While spatial targeting in simulations typically matches measurements with good accuracy, this does not hold for absolute pressure amplitude. Recent work (for example, Krokhmal et al., 2025) shows that even state-of-the-art simulations can deviate from true in vivo pressures by roughly 15% on average due to uncertainties in acoustic properties, skull heterogeneity, and model assumptions. This inherent imprecision limits the resolution with which dose-response relationships can be interpreted.

4) The current manuscript defines stimulation sites in broad anatomical terms ("anterior insula," "dorsal anterior cingulate cortex") but does not provide sufficient detail to establish whether specific functional subdivisions were targeted. The anterior insula comprises dorsal and ventral short gyri with distinct connectivity and functions, while the dACC can be further differentiated into dorsal, rostral, and perigenual/mid-cingulate zones (and should likely be named anterior mid-cingulate cortex). Without clarifying which subregions were stimulated, interpretation of the observed behavioral effects remains somewhat ambiguous. To strengthen anatomical specificity, the authors should include sagittal (and for dACC, also coronal) depictions of the simulated beam profiles relative to key landmarks (insula short gyri, central insular sulcus, frontal operculum; cingulate sulcus and genu).

We thank the reviewer for raising this important point regarding anatomical specificity. We agree that both the aIns and dACC comprise functionally heterogeneous subregions. The region we stimulated in the cingulate cortex is indeed referred to as aMCC in the literature considering the parcellation of the cingulate cortex based on structural considerations, cytoarchitecture and circuitry. However, our target is commonly called dACC in the learning and decision-making literature (see e.g. review by Klein-Flügge et al. Neuron, 2022; Clairis & Lopez-Persem 2023). After discussion with all the co-authors, we decided to stick to dACC as we are concerned that potential readers might missed the connection between our findings and that literature, hence reducing the impact of the decision-making and flexible behaviour community.

We now provide a more precise description of the localisation of the targets in the methods, mentioning the aMCC as described by Palomero-Gallagher et al. (2009), page 5, lines 181-184:

“The dACC was defined anatomically as the region anterior to the anterior commissure, posterior to the genu of the corpus callosum, bounded ventrally by the callosal sulcus. We aimed to stimulate just ventrally of the cingulate sulcus such as to engage the area labelled aMCC by Palomero-Gallagher et al. (2009), with a maximum intensity on region 24c’v. The aIns target was localised in the anterior short gyrus located deep within the Sylvian fissure and bordering the frontal operculum. Supplementary Figure 1 shows a representative participant for the region targeted.”

To address the question of anatomical specificity, we have now changed the Supplementary Table 1 to add localisation information. Supplementary Table 1 provides detailed subject-level information on the simulated acoustic and thermal rise for each stimulation target, including left and right anterior insula and dACC. This table documents peak temperature, pressure, ISPPA, and transcranial mechanical index within a 10 mm spherical mask centred on the intended target, thereby increasing transparency and reproducibility of the stimulation protocol. The table also now reports, for each participant and stimulation target, the MNI coordinates of the peak intensity location identified within a 10 mm spherical ROI centred on the planned target, together with anatomical labels adapted from the AICHA atlas.

Finally, we have added the coronal and sagittal views suggested by the reviewer in the supplementary figure 1.

5) Provide a table of per-subject MNI coordinates (x, y, z) for each target along with acoustic values (on-target pressure etc), enabling readers to evaluate whether stimulation predominantly engaged dorsal vs. ventral anterior insula, or was confined to dorsal cingulate proper vs. rostral/mid-cingulate regions. These additions are necessary to verify anatomical precision and to interpret the functional claims about double dissociations between dACC and aIns. If possible, provide group average pressure maps on an MNI template.

Thank you for this helpful suggestion. As per response to the question (4), we have now added such information, with per-subject MNI coordinates (x, y, z) and anatomical labels for each middle of the pressure field, together with the corresponding acoustic values (including peak pressure within the volume), in Supplementary Table 1. These additions should allow readers to assess whether stimulation predominantly engaged dorsal vs ventral anterior insula and dorsal vs rostral/mid-cingulate regions, and provide a subject-level link between maximal acoustic exposure and anatomical localisation.

6) Acoustic metrics were extracted from 10 mm spheres centered on default MNI coordinates, which may span both dorsal and ventral anterior insula gyri. No per-subject MNI coordinates are provided, preventing evaluation of variability across participants or whether stimulation predominantly engaged dorsal vs. ventral subdivisions of aIns or different dACC subzones.

Acoustic metrics were extracted from 10 mm spheres centered on default MNI coordinates in order to extract peak values in the brain and exclude peak values in the skull and surrounding soft tissues. We now report per-subject MNI coordinates for the reported peak acoustic metrics in Supplementary Table 1.

7) The depth of each individual target from the scalp/transducer exit plane is not reported and should be included in the individual subject tables.

The depth is now reported in the Supplementary Table 2 as ‘steering range (mm)’.

8) The sham used bone-conduction audio to mimic TUS sounds with perceptibility validated in a separate group which is somewhat odd (why not in this group?).

We apologize that this aspect of the sham procedure was not sufficiently clear in the original manuscript. Our sham condition was implemented by playing an audio signal through bone-conduction headphones that mimicked the pulse repetition and timing structure of the real TUS. To create this signal, we generated a waveform matched to the ultrasound protocol in terms of pulse repetition frequency and pulse duration, and we adjusted the sampling rate to approximate the perceptual pitch of the TUS-related sound. Six lab members who had previously experienced the full TUS protocol evaluated several candidate waveforms and selected the one that most closely resembled the real stimulation. This calibration step had to be conducted in a separate group to avoid contaminating expectations in the actual study participants. During the experiment, the audio was played only during the sham condition. After completing all sessions, participants were asked whether they could distinguish between sham and verum stimulation. They could not. We are now reporting the details of how the bone-conduction audio to mimic TUS sounds was created in the methods page 21 in Blinding Procedure.

9) Side-effects appear similar across sessions but still, residual cueing cannot be fully excluded. Please clarify if participants guess condition above chance? Provide a blinding index by session. It is also odd that sensations were queried the next day? Were scalp sensations / jaw vibrations / warmth systematically queried immediately after each session (not only the next day)? Please summarize condition-wise frequencies.

We appreciate the opportunity to clarify this. Side effects were assessed during every session both immediately after the task and again the following day. The next day’s questionnaire was included to capture any delayed side effects, but not to replace the same-day reporting.

10) Stimulation was offline then the task commenced immediately. Provide a brief rationale for the expected window of neuromodulatory after-effects at 500 kHz/10% DC (80 s) and cite prior TUS-human/NHP evidence using similar offline timings. Are effects immediate? If available, report the latency between final pulse and task onset and offset.

We did not systematically record the time between the final pulse of the TUS protocol and the start of the task; however, this latency was typically around 5 minutes (10 minutes maximum). Note that instructions and training were administered before TUS; hence, participants could immediately start with the actual task after the stimulation.

We know write on page 5 lines 147:

“[...] immediately followed (maximal latency: ten minutes) by a motivational Go/NoGo learning task”

And on page 20 lines 776-:

“Directly after the end of the stimulation procedure (maximal latency of ten minutes), participants started the Motivational Go/NoGo Task.”

Please note that, in our results. TUS did not change the response bias (which affects responses immediately from trial 1 onwards), but instead changed two forms of learning biases, whose effects on behaviour are cumulative and only start emerging over time. By their very nature, such effects are most visible in the 2nd half of the task. Hence, we cannot adjust that effects were “immediate”. Instead, they modulated the effects of learning, which only appear over time.

Minor

1) You show M7 (response + learning + persistence bias) best fits sham and dACC. M6 wins aIns but is nested within M7. To solidify conclusions report posterior parameter recoverability (already referenced) and cross-model generalization checks more explicitly in main text (currently in Supplementary). Add a conceptual figure mapping each parameter to behavior (much of this exists in Fig. 2; a single, simplified panel would help general readers).

We now say in the Results section on page 10, lines 383-:

“Model recovery showed that all models were distinguishable from each other, and parameter recovery on M7 showed that all parameters were well identifiable (Supplementary Fig. 6 & 7)”

We also added a conceptual figure in Supplementary Figure 10 that illustrates each parameter by contrasting the characteristic data patterns associated with high versus low bias.

2) The "reset/salience" framing is apt, but several paragraphs could read as over-assigning unique roles to dACC vs aIns. Since both regions co-activate and both active TUS arms shift b/φ_INT, please temper the exclusivity of the claims and emphasize "relative predominance" rather than strict functional segregation.

We have now done this as per our response to the first major point. Particularly the discussion, on page 16, lines 585, now reads:

“Both stimulation conditions also affected Go responses and response persistence in an unspecific manner, though these effects were weak and not significant when correcting for multiple comparisons. Given that dACC and aIns are part of the same resting-state network, the saliency network (Seeley, 2019; Taylor et al., 2008), and regularly co-activate in cognitive tasks (Kurth et al., 2010; Medford and Critchley, 2010), one might expect that stimulating these regions leads to largely similar effects. While this is the case for such unspecific effects, each region also showed a unique effect, suggesting that distinct regions of the saliency network might have distinct roles in learning and response adjustment. Overall, it is likely that both learning processes arise from the interplay of a network of regions, and our results suggest a relative predominance of the dACC and aIns in these processes, respectively. Our study contributes to understanding their different roles in learning from feedback: While the dACC appear important in selecting which environmental signals to use as feedback, the aIns seems important in credit assignment whether these signals were caused by an agent’s own actions.”

3) N=29 is reasonable for within-subject TUS but leaves limited precision for higher-order interactions. Please add a sensitivity analysis (detectable standardized effects for key parameters/contrasts) and precision plots or bootstrap CIs for Δκ and Δφ_DIFF.

We now report a post-hoc sensitivity analysis (power as a function of the value of the effect size Hedges’ g given N = 29) in Suppl. Figure 9.

4) The safety metrics reported are appropriate. Add the exact formula used for MItc (P_neg,tc/√f) and define whether values reported are voxelwise maxima within the ROI vs. global maxima along the beam path.

We have now added the following the methods, page 21, lines 807-:

“The transcranial Mechanical Index (MItc) was computed as MItc = P_neg,tc / √f, where P_neg,tc is the simulated peak rarefactional (negative) pressure after skull transmission (MPa) and f is the ultrasound fundamental frequency in MHz (0.5 MHz). P_neg,tc values were extracted as voxelwise maxima within a 3 mm-radius spherical ROI centred on the stimulation target, likely to include local maxima.”

5) You state ISPPA ≈ 50 W/cm² in water with hydrophone validation. Please give the calibration method (hydrophone model, spatial averaging/aperture, distance), mapping from drive amplitude to on-target pressure, and the assumed attenuation law(s).

Free-field pressures were calibrated in a water tank using an ONDA capsule hydrophone (HGL-series Golden Lipstick) mounted in a low-cost hydrophone tank adapted from Clinard et al., (Ultrasound Med Biol., 2022, 48:157–163) converting voltage from the stable portion of the waveform at focus to pressure using the hydrophone’s calibrated sensitivity to compute peak rarefactional pressure and ISPPA. No attenuation correction was applied for the free-field water calibration beyond the intrinsic frequency-dependent sensitivity correction of the hydrophone (water assumed lossless at 500 kHz). This is reported on page 20, lines 801-.

6) Provide neuronavigation error (FRE/TRE, mean ± SD) and any cross-session repositioning metrics.

We now report the targeting error and distance between the intended and stimulated targets in Table S2. The targeting error is provided by Brainsight, as the distance from the target to the closest point along the line projecting from the transducer’s reference point into the head (along the trajectory) We recorded a single sample, at the midpoint (40 seconds) of the stimulation and report the targeting error for that point. We also report the Euclidean distance between the intended and stimulated target also using the coordinate provided by the Brainsight sampling tool.

Note that we stimulate each target in a single session, therefore we do not have cross-session repositioning metrics.

7) Note counterbalancing / randomization of cue-condition assignments across sessions. Consider reporting any cue-set fixed effects.

We used 3 different orders of sonication conditions (dACC-aIns-sham, aIns-sham-dACC, and sham-dACC-aIns). These orders occurred 10, 9, and 10 times in our sample (total N = 29), suggesting that counterbalancing was optimal. We assessed modelling parameter values were significantly different between these three orders, which was not the case.

Considering cue-set effects, we used different cue sets (different diamond shapes) 1–4 in session #1 (for all participants), cue sets 5–8 in session #2, and cue sets 9–12 in session #3 and in this way achieved counterbalancing of cue sets across conditions. However, this implementation confounds cue-set effects with practice effects across sessions (changes across sessions) and blocks (changes over time within sessions). We now tested for residual cue sets effects using the main effects of session and block as proxies for practice effects as well as their interaction for any non-linear effects that could be indicative of cue-set effects.

There were practice effects on Go/NoGo responses, with participants getting better at the task across sessions (significant interaction between required action and session ID), and the Pavlovian bias getting respectively smaller across sessions (note that the Pavlovian bias is indexed by particular patterns of incorrect responses; as they get overall better at the task, the bias must by definition decrease in size). At the same time, participants’ tendency to repeat responses increased over sessions (which likely is the reason for increased accuracy—participants become better at ignoring infrequent presentations of invalid negative feedback and stick with their response). Beyond such practice effects across sessions, there were no effects of block ID within sessions, nor any interactions between session ID and block ID.

We report this on page 5, lines 162-, page 8, lines 314-, and page 10, lines 390-.

8) Standardize reporting of Cohen's d or Hedges' g for all key contrasts (many are already provided in modelling section).

We now added Hedges’ g, and robust bootstrapped 95%-CIs for all effects of TUS on parameter estimates throughout the manuscript. For mixed-effects models, we already report the regression coefficient b and the 95%-CIs as effect size metrics. Given that all regressors (and outcomes) are z-standardized, these regression coefficients can be interpreted as standardized regression coefficients and compared with each other. Please note that Cohen’s d and Hedges’ g are tied to the performance of a t-test on aggregated data. They are not an appropriate representation of the effects estimated in a mixed-effects model (e.g., a 95%-CI of a Cohen’s d estimate might include 0, while, in the respective mixed-effects model, the effect is strongly significant. Higher power of mixed-effects models is exactly the reason for why we applied them instead of t-tests).

9) Where χ² tests from mixed models are reported, add the test family (Type II/III) and confirm Satterthwaite vs Wald approximations. Without specifying which family and approximation was used, the reported χ² values are ambiguous and not easily reproducible

We now added in the Methods section on page 23 lines 896-:

“We obtained p-values from Wald chi-square tests using Type-3 sums-of-squares via the Anova() function from the car package.”

Please note that we have shared all our code, and analyses are thus reproducible.

10) The Discussion nicely connects biases to psychopathology. Consider adding concrete predictions (e.g., altered κ or φ_DIFF in OCD/MDD) to guide future TUS trials.

We now write in the Discussion section on page 18, lines 718:

“Furthermore, it might open new avenues in treating psychiatric disorders. In particular, in the context of the Motivational Go/NoGo Task, little is known about the underlying learning biases, but they show obvious relationships with psychiatric symptoms: credit assignment processes are frequently found altered in schizophrenia, leading to an underestimation of one’s own and overestimation of others’ agency (Harrow et al., 2009; Voss et al., 2017) . Similar, depression is characterized by a lack of sense of agency/self-efficacy (Bandura, 1977; Maddux and Meier, 1995). In contrast, overly rigid behaviour and perseverative processes are often observed in obsessive-compulsive disorder (OCD), where such symptoms might arise from altered feedback processing or selection of feedback signals(Loosen and Hauser, 2020; Robbins et al., 2012). Such links would have to be corroborated and the efficacy of TUS in treating such symptoms tested in further studies.”

**Reviewer #2 (Remarks to the Author):**

The authors present a well-conducted TUS study to test the effects of sonication of the dACC and anterior insula (aIns) during learning and decision-making, measured with a well-characterised Go/NoGo learning task. The authors have interesting findings, including a dissociation between the behavioural effects of aIns TUS and dACC TUS. There is a robust imaging and sonication protocol, and good evaluation of potential side-effects, which did not differ between the sites or between active and sham stimulation (meaning blinding was sustained and the results are unlikely to be driven by expectations, a critical consideration in intervention studies). Overall, I think it is novel, informative, and cutting-edge from both a methodological and theoretical perspective.

We thank the reviewer for this thoughtful review. We appreciate the recognition of the methodological rigor and are grateful for the acknowledgment of the theoretical contribution and the novelty of using TUS to dissect the computational mechanisms underlying Pavlovian biases. The positive assessment is very encouraging.

I have a few questions/clarifications:

Abstract –

(1)     The authors only introduce Pavlovian response bias in the abstract, making their initial presentation of results less clear (to me, anyway). Instead, I think the authors should briefly describe all three types of Pavlovian biases, if words allow. This would also allow a mention of the null result on response bias, which I think is important enough to merit mention in the abstract given (as I understand it) it was a central part of the authors' hypotheses.

We thank the reviewer for this suggestion and agree. We have now reworded the abstract as follows, first defining all three biases, and then referring to them when reporting the effects of TUS:

“Pavlovian biases reflect how evolutionarily hard-wired tendencies - automatic approach toward reward cues and withdrawal from threat cues - can interfere with flexible, goal-directed action. Such biases arise through three mechanisms: (a) anticipated rewards energize action while anticipated punishments suppress it (response bias), (b) agents learn differently from actions over inactions (learning bias), and (c) reward/punishment cues themselves drive repetitive behaviour, independent of outcomes (perseveration bias).”

And then later:

“Computational modelling revealed a functional differentiation of both regions in Pavlovian biases: while TUS to either region did not affect the response bias, TUS to the aIns decreased people’s learning bias, while TUS to dACC increased participants’ perseveration bias. Although the dACC and aIns are part of the same network and often co-activate during decision-making tasks, TUS interference reveals their distinct roles: the dACC mediates cue-dependence persistence while the aIns is critical for inferring whether outcomes are self-caused.”

Introduction –

(2)      Similarly, an earlier discussion of the authors' original predictions of each TUS condition's effects would be helpful (they are already well-presented in the discussion, specifically the Pavlovian response bias prediction that was not borne out, but this would have been helpful at introduction stage to contextualise the results)

We agree this will contextualise the results. We have now rephrased the introduction, and added the following sections:

“Given that both regions form part of an early "reset" network responding to salient stimuli (Fouragnan et al., 2015) and show rapid valence-related responses (Algermissen et al., 2024, 2022), we predicted that TUS to either region would affect one or more Pavlovian biases. We predicted that TUS to either region would selectively affect action-specific Pavlovian biases, in particular the Pavlovian response bias, defined as the tendency to invigorate action in response to rewards and suppress action in response to punishments. Additionally, based on dACC’s role in behavioural adjustment and processing counterfactual feedback (Fouragnan et al., 2019; Kolling et al., 2012) and on the aIns's role in credit assignment and distinguishing self-caused from externally-caused outcomes (Jabbi et al., 2008; Wittmann et al., 2020), we predicted that perturbing either regions could plausibly alter Pavlovian influences on action selection and learning. We therefore examined whether TUS to dACC or aIns would differentially affect distinct components of Pavlovian control. To this end, we used computational reinforcement learning models designed to isolate these three distinct Pavlovian biases and test our predictions.

We expected TUS to interfere with neural processing in these regions and thus lead to different expression of Pavlovian biases in behaviour (Guitart-Masip et al., 2012; Swart et al., 2017) since both regions have been implicated in different aspects of feedback-based learning, including biased credit assignment (Algermissen et al., 2024; de Boer et al., 2019; Swart et al., 2017). We thus also tested for effects on several Pavlovian biases, including a persistence bias and a learning bias, using computational reinforcement learning models designed to isolate latent cognitive processes.

To foreshadow our results, stimulation of both dACC and aIns altered Pavlovian biases, revealing functional differentiation between these regions. TUS-aIns selectively reduced participants’ tendency to overly attribute rewards to their own actions and their reluctance to attribute punishments to their inactions, captured by a learning bias parameter (Swart et al., 2017). In contrast, TUS-dACC impaired participants’ ability to tell apart the valence of the feedback from the valence of the cue they responded to, captured by a persistence bias parameter (Algermissen et al., 2024). These results indicate dACC relative dominance in selecting which cues in the environment to use as a reinforcer signal, and of the aIns in inferring whether outcomes are self-caused. These two mechanisms —reliance on cue valence as a reinforcer signal and assigning credit to one’s own actions —may be altered in psychiatric disorders (Korn et al., 2014; Shahar et al., 2021) potentially contributing to symptoms such as pathological guilt, externalized blame, and compulsive repetition of actions despite unhelpful outcomes. Disentangling their specific neural contributions is therefore essential for advancing both cognitive neuroscience and clinical psychiatry.”

Methods/Results –

(3) How did the authors deal with effects of TUS condition order (e.g., sham/dACC/aINS versus someone who received dACC/aINS/sham)? This was randomised but did they authors test if there was any effect of condition order on their various outcome measures?

Thank you for this suggestion. We used three different orders of sonication conditions (dACC-aIns-sham, aIns-sham-dACC, and sham-dACC-aIns). These orders occurred 10, 9, and 10 times in our sample (total N = 29), suggesting that counterbalancing was optimal. We assessed whether all of our modelling parameter values (7 parameters) were significantly different between these three orders, which was not the case. We report the fact that we tested for this and that results were non significant on lines 315-, page 9 and lines 398-, page 10.

“As a further control analyses, we tested for effects of session identity (1st, 2nd, 3rd session, using data from all sonication conditions to increase power) on the reported effects. For Go responses, there was an interaction between session ID and required action (χ²(1) = 18.189, p < .001), reflecting that accuracy increased over sessions. For repeat/switch responses, there was a significant main effect of session (χ²(1) = 18.916, p < .001) and a significant interaction between session and past outcome (χ²(1) = 7.898, p = .019): participants overall repeated their responses more often in later sessions and showed a stronger effect of past outcomes on choice repetitions, mostly driven by more repetitions after positive feedback. This pattern might have contributed to higher accuracy in later sessions. We furthermore tested for effects of session order (i.e., order in which all three stimulation conditions were performed) and effects of cue set (i.e., the set of geometric cues which participants saw in a given block), but did not find any effects on Go/NoGo or repeat/switch responses.”

(4)     Similarly, did the authors test or control for effects of sex and age, particularly the latter given the range of 19-59 and previous age-specific effects of certain types of brain stimulation (or was there insufficient power to test this)?

We have now tested for gender and age. We would like to note however, that sampling was not optimised to assess age effects, and, indeed, only 4 participants had an age > 50 years.

In brief, we found an interaction between both gender and age on raw accuracy scores, as well as associated parameter estimates. There were however no significant effects of age or gender on the parameters on which we observed sonication effects, i.e., κ and φ_DIFF, and there were no significant interactions between sonication and gender or age.

We have added these results in the main results at the end of page 8 and page 9:

“As control analyses, we tested for age or gender effect any of the reported effects. For Go/NoGo responses, there were significant interactions between age and required action (b = -0.415, 95%-CI [-0.685, -0.145], χ²(1) = 9.061, p = .003) and gender and required action (b = -0.293, 95%-CI [-0.573, -0.013], χ²(1) = 4.206, p = .040), reflecting effects on task accuracy: Male participants achieved overall higher accuracy than female participants, and younger participants higher accuracy than older participants. For repeat/switch responses, there was a main effect of gender (b = -0.276, 95%-CI [-0.515, -0.038], χ²(1) = 5.157, p = .023) as well as significant interaction between gender and previous outcome (b = -0.310, 95%-CI [-0.528, -0.092], χ²(1) = 7.743, p = .005): Male participants showed overall more repetitions than female participants as well as a stronger effect of past feedback. Specifically, male participants more often repeated responses after positive feedback, which might have contributed to higher performance. Furthermore, there was a significant main effect of age (b = -0.313, 95%-CI [-0.540, -0.085], χ²(1) = 7.250, p = .007), with older participants repeating responses less often than younger participants. This propensity to more often switch responses might have contributed to lower accuracy in older participants.”

And then:

“We again tested for effects of age, gender, session ID, and session order on parameter values (using estimates from all three sonication conditions). Male participants showed overall significantly higher values for the reward sensitivity ρ (χ²(1) = 6.037, p = .014), in line with more deterministic behaviour and higher accuracy. There were no significant effects of age on parameter values. Across session IDs, values of both ρ (χ²(1) = 10.336, p = .006) and ε (χ²(1) = 6.267, p = .044) significantly increased, in line with increasing accuracy across sessions. In consequence, values of π significantly decreased across sessions (χ²(1) = 8.423, p = .015), which is expected since a lower number of errors will lead to a smaller Pavlovian response bias. Finally, there was no effects of session order on any parameter value.”

(5)     Can the authors provide a few more details about their approach to multiple comparisons (only mentioned on p13 in the context of the Go bias parameter and persistence parameters)—does this mean their other parameter results all survived MC correction, or was this only applied to for post-hoc/non-primary comparisons?

This is an important point. We now explicitly state on page 14 lines 511-:

“To correct for multiple comparisons (seven tests for seven parameters), we applied an adjusted alpha-level of .05 / 7 = .007.”

And further down on page 528-:

“Beyond the bias parameters of interest, both TUS conditions also unspecifically increased the Go bias parameter, F(2, 56) = 4.958, p = .010, η2 = 0.071 (Fig. 4C), and the persistence parameter φINT, F(2, 56) = 3.517, p = .036, η2 = 0.055 (Fig. 4E), though neither of these changes was significant when corrected for multiple comparisons. We report follow-up t-tests for completeness.”

(6)     How did the authors plan sample size - what were the statistical power considerations?

We now report a post-hoc sensitivity analysis (power as a function of the value of the effect size Hedges’ g given *N* = 29) in Suppl. Figure 9.

7. Discussion -
(7)     What are the authors' views on the directionality of the effects neurally (i.e., excitatory, inhibitory) on the aIns vs. dACC (and, more speculatively, do they think the neural effects of TUS are consistent across regions with the same protocol, or is it possible it exerts different effects on these different regions due to neuronal morphology/physiology differences)?

The reviewer raises a very interesting question. We now discuss this in the Discussion section on page 19, second and third paragraphs:

“Interestingly, we found similar unspecific effects of TUS-dACC and TUS-aIns on the overall propensity to make Go responses and to repeat previous responses (though not significant when correcting for multiple comparisons), suggesting that sonication of both regions had similar effects in some respects. This observation tentatively suggests that the directionality of the effect (i.e., excitation or inhibition) was the same for both stimulation sites. Previous studies that have used the same stimulation protocol on the posterior cingulate cortex and basolateral amygdala have used magnetic resonance spectroscopy (MRS) to directly measure excitation-inhibition (E/I)-balance in the stimulated regions (Algermissen et al., 2025; Yaakub et al., 2023b). These studies found an inhibitory effect, suggesting that TUS perturbs “normal” activity these regions. Under such a perturbatory influence, one might indeed expect a decrease of the learning bias κ after TUS-aIns, and a failure to inhibit the misleading influence of cue valence after TUS-dACC However, in this study, we did not measure MRS and thus cannot directly test the directionality of the effect on neural activity. Notably, one of the studies using MRS mentioned above (Algermissen et al., 2025) found an excitatory effect of the same protocol in the mid-insula, tentatively suggesting that the same stimulation protocol might have different effects in different regions.

More broadly, it remains an open question whether a given ultrasound protocol induces uniform neural effects across different brain regions. Emerging evidence suggests that this is unlikely to be the case. Differences in local cytoarchitecture, cell-type composition, and baseline functional state may strongly constrain how ultrasound interacts with neural tissue (Newman et al., 2024; Prieto et al., 2020). It is possible that region-specific morphology, circuits and functions shape the functional consequences of ultrasound, even when stimulation parameters are held constant. From this perspective, the effects observed in dACC and aIns may reflect a shared perturbation of task-relevant computations rather than identical cellular mechanisms, highlighting the importance of considering regional and circuit-level context when interpreting the neural impact of TUS. Our findings also highlight the promise of TUS as a neuromodulation tool in cognitive neuroscience. Compared to other methods, such as TMS or transcranial direct current stimulation (tDCS), TUS allows for deeper, more focal targeting of subcortical and midline/lateralised deep cortical structures, making it particularly suited to probing the causal roles of regions like the aIns and dACC. As such, this method opens new possibilities for investigating the neural mechanisms of learning and decision-making. Furthermore, it might open new avenues in treating psychiatric disorders. In particular, in the context of the Motivational Go/NoGo Task, little is known about the underlying learning biases, but they show obvious relationships with psychiatric symptoms: credit assignment processes are frequently found altered in schizophrenia, leading to an underestimation of one’s own and overestimation of others’ agency (Harrow et al., 2009; Voss et al., 2017) . Similar, depression is characterized by a lack of sense of agency/self-efficacy (Bandura, 1977; Maddux and Meier, 1995). In contrast, overly rigid behaviour and perseverative processes are often observed in obsessive-compulsive disorder (OCD), where such symptoms might arise from altered feedback processing or selection of feedback signals(Loosen and Hauser, 2020; Robbins et al., 2012). Such links would have to be corroborated and the efficacy of TUS in treating such symptoms tested in further studies.”
