# Supplementary material for: Ultrasound neuromodulation reveals distinct roles of the dorsal anterior cingulate cortex and anterior insula in learning
Source: PLoS Biol. 2026 May 5;24(5):e3003767. doi: 10.1371/journal.pbio.3003767 (PMC13143107; doi:10.1371/journal.pbio.3003767)
Supplement: Attachment — Submitted filename: review2.docx [file pbio.3003767.s027.docx]

The revised manuscript is substantially improved and addresses most of my prior concerns. In particular, I appreciate that the authors have appropriately softened the original “double dissociation” framing to a more cautious interpretation of functional differentiation / relative predominance, which better reflects the presence of overlapping non-specific effects across active stimulation conditions. The addition of multiplicity-corrected inference, bootstrapped confidence intervals, and nuisance-controlled analyses strengthens the modeling claims, and the revised conclusions are now more consistent with the data.

I find the core conclusion broadly supported: the computational modeling continues to suggest that anterior insula stimulation predominantly alters learning bias (κ), whereas dACC stimulation predominantly alters perseveration bias (φ_DIFF). At the same time, the supplementary cue-wise regressions still show overlapping and often marginal effects across cue types, so the current tempered language is important and should be maintained.

A few points would still improve the manuscript before final acceptance. First, the subject-level dose–response analyses were reportedly performed but omitted because they were null. Even acknowledging uncertainty in simulated in situ pressure, these analyses are directly relevant to causal interpretation and should be shown in supplementary form (e.g., scatterplots or correlation tables relating Δκ, Δφ_DIFF, Δb, and Δφ_INT to simulated pressure/intensity). Second, because the revised interpretation depends on region-specific effects surviving adjustment for shared non-specific components, the nuisance-controlled mixed-model outputs should be presented explicitly (β, CI, p-values) rather than summarized only narratively.

Overall, however, I believe the manuscript is now considerably stronger and that the main conclusions are largely supported provided the interpretation remains cautious.
